# Moving toward Extensively Drug-Resistant: Four-Year Antimicrobial Resistance Trends of *Acinetobacter baumannii* from the Largest Department of Internal Medicine in Slovakia

**DOI:** 10.3390/antibiotics12071200

**Published:** 2023-07-18

**Authors:** Yashar Jalali, Adriána Liptáková, Monika Jalali, Juraj Payer

**Affiliations:** 1 Faculty of Medicine, Comenius University in Bratislava, 5th Department of Internal Medicine, University Hospital Bratislava, Ružinov, Špitálska 24, 813 72, and Ružinovská 4810/6, 821 01 Bratislava, Slovakia; yashar.jalali@fmed.uniba.sk (Y.J.); adamcova.monika@gmail.com (M.J.); 2 Institute of Microbiology, Faculty of Medicine, Comenius University in Bratislava, Špitálska 24, 813 72 Bratislava, Slovakia; adriana.liptakova@fmed.uniba.sk

**Keywords:** *Acinetobacter baumannii*, antibiotic resistance trends, multidrug-resistant, carbapenem-resistant, healthcare-associated infection

## Abstract

*A. baumannii* imposes a great burden on medical systems worldwide. Surveillance of trends of antibiotic resistance provides a great deal of information needed for antimicrobial stewardship programmes nationwide. Clinical data from long-term, continuous surveillance on trends of antibiotic resistance of *A. baumannii* in Slovakia is missing. One hundred and forty-nine samples of *A. baumannii* were isolated over a period of four years. A panel of 19 antibiotics from seven antibiotic categories were tested for the bacterium’s susceptibility. Resistance results were evaluated, and the significance of patterns was estimated using simple linear regression analysis. All isolates were more than 85% resistant to at least 13 out of the 19 tested antibiotics. A significant rise in resistance was recorded for aminoglycosides and imipenem from 2019 to 2022. Colistin and ampicillin-sulbactam have been the only antibiotics maintaining more than 80% efficacy on the bacterium to date. A significant rise in extensively drug-resistant (XDR) strains among carbapenem-resistant (CR) isolates has been recorded. Multidrug-resistance (MDR) among all *A. baumannii* isolates and XDR among CR strains of the bacterium have risen significantly in the last four years.

## 1. Introduction

*Acinetobacter* is a ubiquitous opportunistic coccobacillus, having reservoirs in nearly all environmental niches [1,2,3]. The natural habitats of *Acinetobacter baumannii* are still poorly understood, as it is almost exclusively isolated in close-contact communities and hospital environments [3,4]. Despite being discovered in the early years of the previous century, the bacterium has begun to attract international attention only recently due to a significant rise in isolation of its MDR and even pan-drug-resistant (PDR) strains worldwide [4,5,6]. The bacterium’s strong ability to resist the effects of a wide range of antibiotics, due to both intrinsic and acquired mechanisms, has greatly increased the morbidities and the mortalities caused by *A. baumannii* in recent years [1,4,6,7]. Combined resistance (resistance to fluoroquinolones, aminoglycosides and carbapenems) is a term utilised by the European Centre for Disease Prevention and Control (ECDC) to present epidemiology and trends of drug resistance changes of *Acinetobacter* species [8]. *Acinetobacter* species resistant to one antibiotic in all three tested combined antibiotic families can be regarded as MDR species. The percentage of combined resistance isolates of *Acinetobacter* species, in the last published surveillance by the ECDC in 2021, varies widely in Europe, from 7% to above 50% (most intensive in southern and eastern European countries) [8]. The rise in the percentage of combined resistant isolates of the *Acinetobacter* species in Slovakia from 24% in 2020 to 53% in 2021 is alarming [8].

Resistance to carbapenem alone is considered to be a marker of extensive antimicrobial resistance, since it involves a large range of co-resistance to other unrelated antibiotic classes [6]. Six years after the listing of CR *A. baumannii* by the World Health Organisation (WHO) as a critical priority bacterium for which new antibiotics are urgently needed, no real development has occurred [1,4,7,9]. The rate of clinical outbreaks of CR *A. baumannii* ranges between 1% and over 30% in Europe, being again most intense in eastern and south-eastern countries [1].

Slovakia’s location in a region with some of highest percentages of combined resistant isolates of *Acinetobacter* species (Hungary: 80%, Romania: 89%, Poland: 67%, Czechia: 50%, Croatia: 98%) makes the surveillance of trends of the bacterium’s antibiotic resistance of great importance [8]. This data provides insight into the expected prevalence and trends of the antibiotic resistance of *A. baumannii*, which is needed to develop the rationale for antibiotic stewardship policies battling against the bacterium not only in Slovakia but the whole region.

This study presents comprehensive data from four years of surveillance (2019 to 2022) on trends of antibiotic resistance of all clinically relevant *A. baumannii* isolates from the largest department of internal medicine in Slovakia.

## 2. Results

### 2.1. Patients Characteristics and Demographic Data

A total of 11,856 patients were hospitalised during the study period in our department (3618 patients in 2019, 2940 patients in 2020, 2579 patients in 2021, and 2719 patients in 2022). A total of 149 samples of *A. baumannii* were isolated. Four samples were isolated from patients hospitalised in intensive care unit (ICU) (4/149, 3%), 13 samples were isolated from patients hospitalised in intermediary ward for critically ill patients (13/149, 9%) and 132 samples were isolated from patients hospitalised in standard male and female wards (132/149, 88%) during study period. Average hospitalisation in ICU was recorded seven to ten days, and in other wards 25 days. Male patients were more likely to be infected by *A. baumannii* than female patients (57% to 43%). The mean age of male patients was 12 years higher than that of female patients (Table 1).

The prevalence of *A. baumannii* was 0.8% in 2019, 1% in 2020, 2% in 2021, and 1.5% in 2022. MDR trends changed from 32% in 2019 to 43% in 2020 due to an increase in resistance to tobramycin among isolated strains. This trend further changed to 67% in 2021 and 53% in 2022 (Table 1). There was a significant association between the trend of prevalence of the bacterium and its MDR (R^2^ = 0.97, *p* = 0.01 confidence level (CL): 95%). The total healthcare-associated infection (HAI) rate was 85% over the four years. We recorded a steady rise in the percentage of HAI, from 60% in 2019 to 90% in 2022. Despite similarities in patterns of HAI and prevalence of the bacterium (Table 1), there was no statistically significant association between them (R^2^ = 0.61, *p* = 0.21 CL: 95%). The associated mortality rate (AMR), defined as the effect of the infection on patient death as an outcome, in association with other comorbidities, is shown in Table 1. The AMR percentage (number of diagnosed patients with *A. baumannii* who died during the same hospitalisation without laboratory data suggestive of infection management divided by the number of all diagnosed cases of *A. baumannii*, expressed as a percentage) was 40% in total, meaning 40% of patients diagnosed with the bacterium died during the same hospitalisation. There were no associations between AMR and the MDR trend, prevalence, and/or HAI.

### 2.2. Distribution of Samples’ Isolation

**Figure 1:**
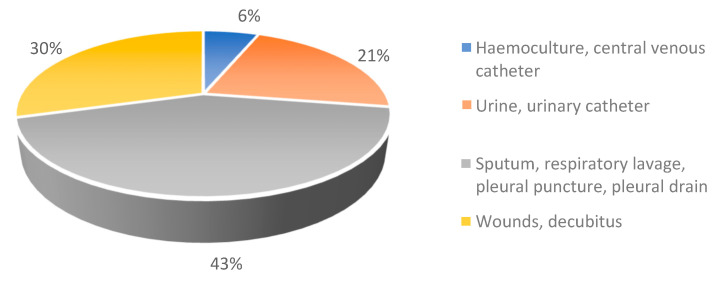
Frequency of isolation (%) of *A. baumannii* from a given site.

*A. baumannii* was predominantly isolated from respiratory secretions (n: 64/149, 43%), followed by wounds or decubitus samples (n: 44/149, 30%), urinary tract infections (n: 32/149, 21%), haemocultures, and samples from central venous catheter-related infections (n: 9/149, 6%) (Figure 1).

### 2.3. Demographic Data and Prevalence of Carbapenem Resistant Isolates

The number of CR strains among all isolates, despite an initial rise during the years 2019 and 2020 (68% to 75%, respectively), declined to 39% and 44% in 2021 and 2022, respectively. The percentage of multidrug-resistant cases hovered steadily below 25% among all CR isolates. However, alarmingly, the number of extensively drug-resistant strains rose continuously among CR isolates, reaching 83% of cases in the year 2022 (Table 2).

We recorded an increase in the rate of HAI from CR strains during the study period. The association between the increase in percentage of HAI and the percentage of XDR isolation was statistically significant (R^2^ = 0.97, *p* = 0.01, CL: 95%), meaning the higher the percentage of HAI, the higher the probability of isolation of the XDR bacterium among CR strains.

### 2.4. Trends of Antibiotic Resistance

A susceptibility test was performed on the panel of 19 antibiotics from seven antibiotic families as the routine antibiogram examination on the bacterium. The antibiotic families included penicillins (ampicillin, ampicillin-sulbactam, and piperacillin-tazobactam), cephalosporines (cefotaxime, ceftazidime, cefuroxime, cefepime, and cefoperazone-sulbactam), carbapenems (imipenem, ertapenem, and meropenem), monobactams (aztreonam), fluroquinolones (ciprofloxacin), aminoglycosides (amikacin, gentamicin, and tobramycin), tetracyclines (tetracycline), sulphonamides (trimethoprim-sulfamethoxazole), and polymyxins (colistin).

**Figure 2:**
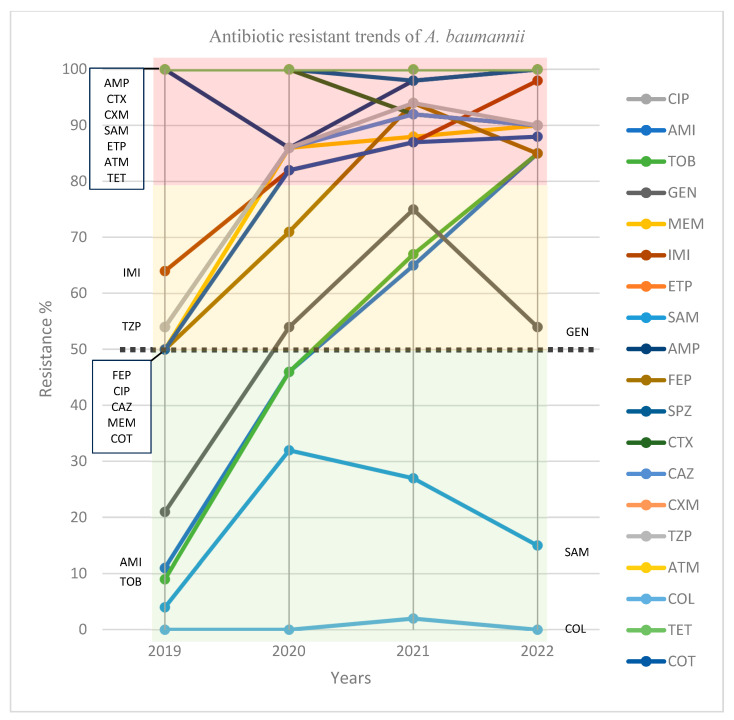
Antibiotic resistance trends of *A. baumannii*. AMI: amikacin, AMP: ampicillin, ATM: aztreonam, CAZ: ceftazidime, CIP: ciprofloxacin, COL: colistin, COT: co-trimoxazole (trimethoprim-sulfamethoxazole), CTX: cefotaxime, CXM: cefuroxime, ETP: ertapenem, FEP: cefepime, GEN: gentamycin, IMI: imipenem, MEM: meropenem, SAM: ampicillin-sulbactam, SPZ: sulperazone (cefoperazone-sulbactam), TET: tetracycline, TOB: tobramycin, TZP: tazocin (piperacillin-tazobactam).

All isolated strains of *A. baumannii* showed 100% resistance to ertapenem, aztreonam, tetracycline, and cefuroxime. Resistance to ampicillin, cefotaxime, and cefoperazone-sulbactam was steadily above 90% (Figure 2 and Table 3). Resistance to aminoglycosides (amikacin and tobramycin) showed a significant rise from 11% in 2019 to 85% in 2022 for amikacin (R^2^ = 0.97, *p* = 0.01) and from 9% in 2019 to 85% in 2022 for tobramycin (R^2^ = 0.96, *p* = 0.01). Although resistance to gentamycin doubled (from 21% in 2019 to 54% in 2022) in the period of four years (Figure 2 and Table 3), it was not a statistically significant increase (R^2^ = 0.48, *p* = 0.30). The increase in antibiotic resistance continued for all tested antibiotics in the penicillin, cephalosporine, and carbapenem families with the exception of ampicillin-sulbactam. The same increase in resistance was recorded in all isolated cases for ciprofloxacin (from 54% in 2019 to 90% in 2022, R^2^ = 0.68, *p* = 0.17) and trimethoprim-sulfamethoxazole (from 50% in 2019 to 88% in 2022, R^2^ = 0.72, *p* = 0.14). However, none of the trends of resistance in the tested antibiotics (except for imipenem, amikacin, and tobramycin), despite a positive linear projection plotted on the graph, was statistically significant (Table 3, Figure 2). Data regarding antibiotic resistance trends for separate antibiotic categories are provided in Supplementary File S1.

The significance of resistance trends for antibiotics with horizontal projection observed on the graph was not calculated.

### 2.5. Trends of Antibiotic Resistance among CR Isolates

**Figure 3:**
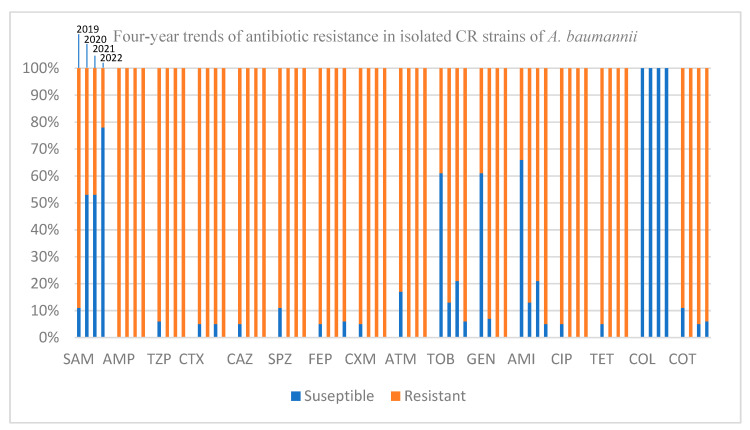
Four-year trends of antibiotic resistance in isolated CR strains of *A. baumannii*. AMI: amikacin, AMP: ampicillin, ATM: aztreonam, CAZ: ceftazidime, CIP: ciprofloxacin, COL: colistin, COT: co-trimoxazole (trimethoprim-sulfamethoxazole), CTX: cefotaxime, CXM: cefuroxime, FEP: cefepime, GEN: gentamycin, SAM: ampicillin-sulbactam, SPZ: sulperazone (cefoperazone-sulbactam), TET: tetracycline, TOB: tobramycin, TZP: tazocin (piperacillin-tazobactam).

Four years of antibiotic resistance trends for isolated CR strains of *A. baumannii* are shown in Figure 3. Four columns are displayed above each tested antibiotic, with each representing a year from 2019 to 2022. Accumulated percentage of susceptibility (in blue) is shown in contrast to accumulated percentage of resistance (in orange) to a given antibiotic in a given year for each column.

All isolated CR strains of the bacterium showed 100% resistance to ampicillin, piperacillin-tazobactam, cefotaxime, ceftazidime, cefoperazone-sulbactam, cefuroxime, aztreonam, gentamycin, ciprofloxacin, and tetracycline in 2022 (Figure 3). Susceptibility to the rest of the tested antibiotics (except for ampicillin-sulbactam and colistin) was less than 10% (Figure 3), reflecting the 83% XDR phenotype of all CR isolates of the bacterium in this year. Resistance to carbapenems (as expected) was strongly associated with the XDR phenotype (64% vs. 3%, *p* = 0.001).

## 3. Discussion

This study provides the first clinical data from a long-term continuous surveillance programme on epidemiology, HAI, associated mortality rate, and trends in antibiotic resistance of *A. baumannii* from Slovakia.

A high percentage of HAI was recorded in our study. Nearly 85% of all samples were isolates from patients who acquired the bacterium during their current hospitalisation. The association between duration of hospitalisation and increased risk of acquiring HAI has been demonstrated in several publications [10,11,12]. It is fairly widely accepted that the longer the patient is hospitalised, the higher their chance of contracting a HAI. Hence, minimum average duration of hospitalisation until diagnosis of HAI with a given bacterium is of great epidemiological and clinical importance. In our previous published report on the association between HAI *A. baumannii* infection and duration of hospitalisation (from July 2019 to October 2020), we demonstrated that an average of 14 days’ hospitalisation was associated with elevated risk of being infected with strains of the bacterium in our department [13]. This trend was similar both in MDR and CR isolates [13]. In the current study, however, patients were hospitalised for 25 days on average before being diagnosed with *A. baumannii*-related infections. The difference between these two data could possibly be explained by the larger cohort of patients in the current study. Since data from epidemiological studies on the state of HAIs of *A. baumannii* from a nationwide database or other single-centre studies in Slovakia are missing, optimisation of this average based on a comparison of available data is not possible for the authors. However, similar to our data, an average of 15 days (or longer) of hospitalisation prior to isolation of *A. baumannii* has been demonstrated in studies from other countries [14,15,16].

In several studies, the presence of HAI has been shown to significantly contribute to all-cause mortality with a non-significant odds ratio [16,17,18,19]. These data suggest that the higher mortality rate in HAI isolates is associated with the higher prevalence of virulent phenotypes due to increased antimicrobial treatment exposure [16]. Alrahmany et al., demonstrated in a recent study that prolonged hospitalisation of more than seven days was a significant predictor of higher mortality rate in *A. baumannii*-related infections (*p* < 0.05) [16]. They argued that 65% of patients who were diagnosed with MDR *A. baumannii* infection during hospitalisation were hospitalised on average for more than seven days, significantly contributing to the outcome of the disease. These data correlate with our findings, demonstrating a significant association between HAI and isolation of XDR strains (R^2^ = 0.97, *p* = 0.01, CL: 95%). As prolonged hospitalisation is an important contributor to the increased cost associated with infection complications, efforts to minimise the nosocomial transmission of HAI should be encouraged. Therefore, such HAI-reducing efforts, along with the resultant decreased mortality and cost, also provide the benefits of reduction in risk of transmission of other MDR organisms and the use of broad-spectrum antibiotics.

In addition to prolonged hospitalisation and HAI, several other risk factors have been identified for the acquisition of antibiotic-resistant *A. baumannii*. Some of these risk factors include prior colonisation with the bacterium, invasive procedures, and ICU stay [20,21,22].

Since only 4 of 149 (3%) of samples were isolated from patients hospitalised in the ICU, an association between ICU hospitalisation and acquisition of *A. baumannii*, unlike the mentioned reports [23,24,25], was not demonstrated in our study. Lack of such a relationship could be explained by the relatively low percentage (34%) of bacterium isolation associated with prior invasive procedures (in total), and the short average duration of ICU hospitalisation in the department (average of seven to ten days).

Urinary catheterisation (UC) along with central venous catheterisation (CVC) were the two most common invasive procures associated with acquisition of *A. baumannii* infection in our study (nearly 34% of cases). All patients from whom the bacterium was isolated from urinary tract infections (UTI) also had UC at the time of sampling (30% of total isolated cases). Hence, UC was the most important invasive procedure considered a risk factor for acquisition of *A. baumannii* infection. Six out of nine isolated blood-borne *A. baumannii* infections were associated with CVC (4% of total isolated cases). Although the highest number of samples (43%) were isolated from respiratory tract-related infections, no invasive ventilation was recorded for any of the patients.

Due to the defined exclusion criteria of the study, an evaluation of association of prior colonisation with the bacterium was not performed.

Old age is generally associated with higher numbers of comorbidities, immunocompromised status, prolongation of hospitalisation, and increased disease severity. The predominance of infection by *A. baumannii* bacteria in males (57% compared to 43% in females, Table 1), in this sense, could be partly explained by the age difference between the male and female patients (mean of 12 years), which included an older cohort of male patients.

Several statistically significant changes in antibiotic resistance rates of *A. baumannii* were observed between January 2019 and December 2022. All isolates of *A. baumannii* were nearly 100% resistant to tetracycline, aztreonam, ertapenem, cefoperazone-sulbactam, and cefuroxime throughout the study period. By the end of 2022, more than 85% resistance was recorded in the other 11 tested antibiotics. Although statistically, the model of resistance pattern of these antibiotics with positive linear trajectory did not fit the confidence level, the percent of susceptibility of the bacterium was recorded at less than 15% for all of them. A significant rise in the pattern of resistance was recorded for amikacin (R^2^ = 0.97, *p* = 0.01), tobramycin (R^2^ = 0.96, *p* = 0.01), and imipenem (R^2^ [26] = 0.94, *p* = 0.02). An exception to this rising resistance pattern was recorded only for colistin (0% resistance), ampicillin-sulbactam (15% resistance), and gentamycin (54% resistance), which are known as last-resort antibiotics left as treatment options for the bacterium [27,28].

An annual growth of 4% in broad-spectrum antimicrobial consumption in 2021 alone in Slovakia (surveillance report of antimicrobial consumption in EU/EEA issued by ECDC) places the country among the worst in Europe (just after Poland, Greece, and Croatia) [29]. Demonstrating the extent and rate of resistance changes of *A. baumannii* in light of broad-spectrum antibiotic consumption growth is worrisome, emphasizing the urgent need for a stricter nationwide antimicrobial stewardship programme.

To date, the only epidemiological data regarding the antibiotic resistance of *A. baumannii* in Slovakia can be acquired from a surveillance atlas of infectious disease published by the ECDC [8]. These data, however, have their limitations. Firstly, the estimate of national population coverage (for Slovakia in the last published report, this was 56%) does not include *A. baumannii* due to outliers in some countries [30]. Hence, the estimation of the epidemiological significance of the presented percentage for smaller or larger samples is unclear. Secondly, it provides information on the resistance of the bacterium to only three antibiotic families (aminoglycosides, fluroquinolones, and carbapenems). As our data demonstrated, susceptibility of the bacterium to ampicillin-sulbactam, for example, not only remained low but showed a decrease in resistance trend in last two years (Figure 2). Such clinically important information cannot be obtained from the ECDC report.

Our data demonstrates that the MDR of *A. baumannii* nearly doubled in the last four years in our department. Importantly, the percentage of MDR isolates in our cohort of patients was higher both in 2020 and 2021 in comparison with national data obtained from the surveillance atlas of infectious disease published by the ECDC (data from 2022 have not yet been published) [8]. This difference could likely be due to inappropriate antibiotic utilisation or cross-acquisition of resistance in our department. There was a significant association between patterns of MDR and prevalence of the bacterium (R^2^ = 0.97, *p* = 0.01 confidence level (CL): 95%). This means that an increase of antibiotic resistance in the bacterium could predict an increase in its prevalence in the future. In a recent study on the prevalence of *A. baumannii* published by Chaoying Ma et al. [1], using data from the European Antimicrobial Resistance Surveillance Network (EARS-Net) and the Central Asian and European Surveillance of Antimicrobial Resistance Network (CAESAR) from the WHO, it was demonstrated that the relation between prevalence and resistance can be explained by the exponential regression model. The authors stated that once antibiotic resistance rates are low, the isolation rate of the bacterium is expected to be stable, but once the resistance rate increases by 50% or more, a surge in prevalence could be expected. Although we did not record the expected surge in the percentage of prevalence in our study (perhaps due to the monocentric nature of the study and small sample size), a clear association between the two variables was demonstrated.

Because of its great importance in clinical practice (due to involvement of large range of co-resistance to other unrelated antibiotic classes), CR *A. baumannii* has been the centre of attention in research on antibiotic resistant trends of the bacterium in recent years. Due to this importance, we performed separate analyses on the antibiotic resistance data of CR isolates of *A. baumannii* in our study. Although numbers of CR isolates were fairly stable (on average 52% of cases over the four years) and prevalence of CR strains stayed below 1%, the percentage of XDR strains among CR isolates rose significantly nonetheless, showing a significant association between CR and XDR phenotypes (64% vs. 3%, *p* = 0.001). Since resistance to carbapenems is considered to be a marker of extensive antimicrobial resistance [31,32,33], this finding was not surprising. However, the speed of development of the XDR phenotype (from 47% in 2019 to 83% in 2022) among CR isolates is worrisome. It is noteworthy to mention that all isolated XDR strains were CR. The pattern of resistance over four years changed in such a way that the bacterium’s susceptibility to amikacin and tobramycin dropped to less than 10% and susceptibility to eight other antibiotics dropped to 0% (Figure 3), marking the rapid movement of CR strains toward being completely XDR in the coming years. In a recent study published by Appaneal et al., on a cohort of 4599 patients diagnosed with *A. baumannii* infection from a nationwide database in the USA [34], diagnosis with *A. baumannii* was stated as an indicator of poor outcome in total for all patients. Moreover, they explained that poor outcome was significantly associated with MDR and/or CR infection phenotypes of the bacterium. The associated mortality rate of the bacterium in our study stayed at around 40%, in accordance with data published by Appaneal et al., However, although slightly higher, the associated mortality rates of CR and non-CR isolates were not significantly different (40% vs. 46%).

Since genome-based strain typing and whole-genome sequencing data on the antibiotic resistance of *A. baumannii* strains in Slovakia is still unavailable, we may only estimate the diversity of the gene pool the bacteria might have. Several studies have demonstrated horizontal gene transfer of mobile antimicrobial-resistant genetic elements (known as mobile gene element MEG) to and from *A. baumannii* through plasmids, integrons, insertion sequences, transposons, prophages, etc. [35,36,37,38]. *K. pneumoniae* is one of the most well-studied bacteria, known as a main pool of drug resistance and as a transporter of resistance genes among Gram-negative bacteria, including *A. baumannii* [37,39,40]. In a recent study published by Koreň et al., a whole-genome sequencing of CR strains of *K. pneumoniae*, isolated from the Bratislava University Hospital complex, was performed [41,42]. The results indicated a high presence of NDM-1, SHV-11, SHV-168, CTX-M-15, OXA-1, and KPC-2-encoded serine carbapenemase genes responsible for the carbapenemase activity of isolated strains. That could only serve as an indication of the antibiotic-resistant genetic pool and MGEs of circulating *A. baumannii* strains in the same healthcare facility.

Limited available antimicrobial treatment along with growing resistance to scarce available options make *A. baumannii* one of the most important topics of debate in clinical microbiology today. As demonstrated from our as well as others’ data, colistin remains the single most effective antibiotic used in the treatment of MDR strains of *A. baumannii*. However, the emergence of colistin-resistant strains of the bacterium poses an extreme danger to healthcare systems worldwide. In a study published by Nowak et al., an alarmingly high incidence of pan-drug-resistant *A. baumannii* isolates were collected from ventilator-associated pneumonia in Greece, Italy, and Spain [43]. In their study, 45% of 65 clinically isolated strains were resistant to colistin. To overcome such complicated and severe infection, several enhancement methods, including increased colistin loading dose, higher maintenance dose, and combination therapy with other antibiotics, have been implemented [44]. As an example, the combination of colistin with nicodamids has been shown to be an effective treatment for *A. baumannii* colistin-resistant strains [45]. The interaction of nicodamids with the negatively charged outer membrane of colistin-resistant strains leads to a synergistic effect with colistin. Several other studies have suggested combination use of colistin with meropenem due to improvement in minimum inhibitory concentration of antibiotics in treatment of CR strains of the bacterium [46,47]. Despite the data demonstrating the benefit of drug combination therapy in cases of carbapenem- and colistin-resistant strains of the bacteria, recent studies tend to favour the single-treatment regimen or monotherapy due to its lower mortality rates and shorter length of stay (LOS), explained by the limited development of antibiotic-related adverse events. A meta-analysis published by Huang et al., demonstrated that colistin monotherapy was associated with similar rates of clinical improvement, drug-related toxicity, and hospital mortality in comparison to combination therapy with meropenem. They stated that combination therapy with meropenem was not superior to monotherapy with colistin. Similar data were recently published by other researchers. [47,48]. That is why the optimisation of treatment of such strains, especially due to their high mortality rate, and utilisation of best treatment method are widely open to debate and currently uncertain. As antibiotics are not part of chronic treatment regimens, large pharmaceutical companies have little interest in investing in new antibiotics [49]. Hence, drug repurposing strategies seem to be a better solution for the time being, even with their limitations. However, the combination of repurposed drugs with existing antibiotics may result in an increase of toxic side effects and interfere with the pharmacokinetics, pharmacodynamics, solubility, and conservation of the respective antibiotic [50]. That is why the need for new molecules is more urgent than ever. Until then, these results emphasise the extreme importance of routine environmental disinfection, staff hygiene, minimisation of invasive procedures or their implementation duration, optimisation of hospitalisation period in relation to age and comorbidities, and utilisation of a rational, strict antibiotic stewardship programme.

## 4. Materials and Methods

### 4.1. Healthcare Facilities, Patients, and Specimens

In this observational, prospective, cross-sectional study, we monitored and gathered data from all clinically relevant isolates of *A. baumannii* from patients hospitalised in two standard male and female wards, one an intermediary ward for critically ill patients with need of central monitoring and the other an intensive care unit of the fifth department of internal medicine of University Hospital Bratislava, Ružinov for a period of four years (from the beginning of 2019 to the end of 2022). The majority of the hospitalised patients were from the Bratislava region. To the best of our knowledge, our data (in whole or in part) were not used in production of any ECDC reports on antibiotic resistance of the bacterium during the study period. For each isolate, we collected information on place of sampling, antibiogram, whether the sample was collected from a HAI or non-healthcare associated infection (NHAI), and whether the patient was discharged or deceased during our monitoring. There was no specific process for the selection of patients in this study. However, all patients included in the study had active infectious processes caused by *A. baumannii* with raised inflammatory markers. Patients with a prior history of colonisation with *A. baumannii* were excluded from the study.

### 4.2. Isolation and Proof of Bacterial Strains

Sterile swabs were used to collect samples from the nose, tonsils, wounds, and decubitus. Blood sampling was performed via peripheral intravenous puncture. Urine was collected via mid-stream urine sampling or via urinary catheter. Samples were processed using conventional methods approved by the Slovak Ministry of Health. Strain identification was performed in accordance with classical isolation and classical bio-chemical and cultivation methods with a Bruker MALDI Biotyper (Bruker, Billerica, MA, USA), which uses the MALDI-TOF Mass Spectrometry method [51]. Antibiotic testing results were interpreted according to EUCAST guidelines (European Committee on Antimicrobial Susceptibility Testing). The panel of tested antibiotics was pre-determined by diagnostic laboratories, defined for Gram-negative isolates.

### 4.3. Statistical Analysis

Data collected during the monitoring period were analysed using Microsoft Excel. A simple linear regression test was used to calculate the significance of the antibiotic resistance trend over time. *p* value was determined using a *t* distribution with n − 2 degrees of freedom (*df*) (in a simple linear regression sample test). The confidence level of the *p* value was determined as 95%. P^1^ as true prevalence was defined as the number of identified infected individuals with *A. baumannii* hospitalised in the department divided by the total number of hospitalised patients in the department for a given year. P^2^ as CR prevalence was defined as the number of CR isolates of *A. baumannii* divided by the total number of hospitalised patients in the department for a given year. The combined resistant rate for *A. baumannii* was defined as the accumulated percentage of resistance of the bacterium to three categories of antibiotics (namely: fluoroquinolones, aminoglycosides, and carbapenems) in a given year. MDR was defined as the non-susceptibility of the bacterium to ≥1 agent in ≥3 antimicrobial categories. XDR was defined as non-susceptibility to ≥1 agent in all but ≤2 antimicrobial categories.

### 4.4. Ethical Consent

Ethical review and approval were not required for the study (as an observational study) on human participants in accordance with local legislation and institutional requirements. Written informed consent for participation was not required for this study in accordance with national legislation and the institutional requirements.

## 5. Conclusions

Multidrug resistance among all *A. baumannii* as well as extensive drug resistance among carbapenem-resistant strains of the bacterium has risen significantly over the last four years in our department. MDR is significantly associated with the prevalence of the bacterium, and with the current rising pattern of resistance, an increase in the prevalence of *A. baumannii* is expected in the coming years. Colistin, ampicillin-sulbactam, and to a much lesser extent gentamycin are the only antibiotics left to have efficacy on isolated strains of *A. baumannii*. No colistin-resistant strain of *A. baumannii* was isolated. Prolonged hospitalisation is associated with an increase in HAI rates. The significant association of HAI with the isolation of XDR strains, along with high rate of associated mortality of the bacterium, could be a predictor of poor outcome. These results emphasise the urgent need for new treatment options, optimisation of antibiotics stewardship programmes, and stricter epidemiologic control and management of the spread of the bacterium’s infection.

## Figures and Tables

**Table 1:** Number of isolated *A. baumannii*, prevalence, multidrug-resistance, and extensively drug-resistant traits.

	Total	2019	2020	2021	2022
Number of hospitalised patients	11,856	3618	2940	2579	2719
Number of isolated *A. baumannii*	149	28	28	52	41
Number of male patients (n/N, cases %)	85/149, 57%				
Age of male patients (Mean)	72.7 (SD ± 18.2)				
Number of female patients (n/N, cases %)	64/149, 43%				
Age of female patients (Mean)	60.2 (SD ± 20.2)				
Hospitalisation duration (Av. days)	25.3				
Healthcare-associated infection (n/N)	126/149	17/28	24/28	48/52	37/41
Cases (%)	85%	60%	85%	92%	90%
Associated mortality rate (n/N)	61/149	11/28	12/28	23/52	15/41
Cases (%)	40%	39%	43%	44%	37%
Prevalence (P^1^ %)		0.8%	1%	2%	1.5%
Number of MDR strains among all isolates (n/N)	78/149	9/28	12/28	35/52	22/41
Cases (%)	52%	32%	43%	67%	53%
Number of XDR strains among all isolates (n/N)	50/149	9/28	12/28	14/52	15/41
Cases (%)	33%	32%	42%	26%	36%

Av.: average, N: total number of isolated samples, n: number of isolated samples for given variable (for example: healthcare-associated infection (n/N) means number of isolated healthcare-associated infection samples to total number of isolated samples, also presented in percent form), P^1^: true prevalence. SD: standard deviation, XDR: extensively drug-resistant.

**Table 2:** Carbapenem-resistant strains of *A. baumannii*, their prevalence, and resistant traits.

	Total	2019	2020	2021	**2022**
Number of CR strains among all isolates (n/N)	78/149	19/28	21/28	20/52	18/41
Cases (%)	52%	68%	75%	39%	44%
Prevalence of CR isolates (P^2^ %)		0.5%	0.7%	0.7%	0.6%
Healthcare-associated infection among CR isolates (n/N)	62/78	12/19	16/21	17/20	17/18
Cases (%)	79%	63%	76%	85%	94%
Associated mortality rate among CR isolates (n/N)	36/78	9/19	10/21	10/20	7/18
Cases (%)	46%	50%	48%	50%	39%
Number of MDR cases among CR isolates (n/N)	15/78	4/19	3/21	5/20	3/18
Cases (%)	19%	21%	14%	25%	16%
Number of XDR strains among CR isolates (n/N)	50/78	9/19	12/21	14/20	15/18
Cases (%)	64%	47%	57%	70%	83%

CR: carbapenem-resistant, MDR: multidrug-resistant, N: total number of isolated samples, n: number of isolated samples for given variable (for example: healthcare-associated infection (n/N) means number of isolated healthcare-associated infection samples to total number of isolated samples, also presented in percent form), P^2^: carbapenem-resistance prevalence, XDR: extensively drug-resistant.

**Table 3:** Antibiotic resistance trends of all isolated *A. baumannii* from 2019 to 2022.

	2019	2020	2021	2022	R^2^-*p* (CL: 95%)
ATB	No. R/S (%)	No. R/S (%)	No. R/S (%)	No. R/S (%)	
Ampicillin	28/0 (100%)	24/4 (86%)	51/1 (98%)	41/0 (100%)	-
Ampicillin-sulbactam	1/27 (4%)	9/23 (32%)	14/38 (27%)	6/34 (15%)	-
Piperacillin-tazobactam	15/13 (54%)	24/4 (86%)	47/5 (94%)	37/4 (90%)	0.67–0.18
Cefotaxime	28/0 (100%)	28/0 (100%)	48/4 (92%)	37/4 (90%)	-
Ceftazidime	15/13 (54%)	24/4 (86%)	48/4 (92%)	37/4 (90%)	0.68–0.17
Cefuroxime	28/0 (100%)	28/0 (100%)	52/0 (100%)	41/0 (100%)	-
Cefepime	14/14 (50%)	20/8 (71%)	49/3 (94%)	35/6 (85%)	0.74–0.13
Cefoperazone-sulbactam	28/0 (100%)	28/0 (100%)	51/1 (98%)	41/0 (100%)	-
Imipenem	18/10 (64%)	23/5 (82%)	45/7 (87%)	40/1 (98%)	0.94–0.02
Meropenem	14/14 (50%)	24/4 (86%)	46/6 (88%)	37/4 (90%)	0.68–0.17
Ertapenem	28/0 (100%)	28/0 (100%)	52/0 (100%)	41/0 (100%)	-
Aztreonam	28/0 (100%)	28/0 (100%)	52/0 (100%)	41/0 (100%)	-
Ciprofloxacin	15/13 (54%)	24/4 (86%)	48/4 (92%)	37/4 (90%)	0.68–0.17
Amikacin	3/15 (11%)	13/15 (46%)	34/18 (65%)	356 (85%)	0.97–0.01
Tobramycin	3/25 (9%)	13/15 (46%)	35/17 (67%)	35/6 (85%)	0.96–0.01
Gentamycin	6/22 (21%)	15/13 (54%)	3913 (75%)	22/19 (54%)	-
Tetracycline	28/0 (100%)	28/0 (100%)	52/0 (100%)	41/0 (100%)	-
Trimethoprim sulfamethoxazole	14/14 (50%)	23/5 (82%)	45/7 (87%)	36/5 (88%)	0.72–0.14
Colistin	0/28 (0%)	0/28 (0%)	2/49 (4%)	0/41 (0%)	-

ATB: antibiotic, No: number, R: resistance, S: susceptibility, R^2^: R square (coefficient of determination), CL: confidence level.

## Data Availability

Not applicable.

## Supplementary Materials

The following are available online at https://www.mdpi.com/article/10.3390/antibiotics12071200/s1, Supplementary File S1: Antibiotic resistant trends of *A. baumannii*. AMI: amikacin, AMP: ampicillin, ATM: aztreonam, CAZ: ceftazidime, CIP: ciprofloxacin, COL: colistin, COT: co-trimoxazole (trimethoprim-sulfamethoxazole), CTX: cefotaxime, CXM: cefuroxime, ETP: ertapenem, FEP: cefepime, GEN: gentamycin, IMI: imipenem, MEM: meropenem, SAM: ampicillin-sulbactam, SPZ: sulperazone (cefoperazone-sulbactam), TET: tetracycline, TOB: tobramycin, TZP: tazocin (piperacillin-tazobactam).
