# Supplementary material for: Moving toward Extensively Drug-Resistant: Four-Year Antimicrobial Resistance Trends of Acinetobacter baumannii from the Largest Department of Internal Medicine in Slovakia
Source: Antibiotics (Basel). 2023 Jul 18;12(7):1200. doi: 10.3390/antibiotics12071200 (PMC10376473; doi:10.3390/antibiotics12071200)
Supplement: Supplementary file 1 [file antibiotics-12-01200-s001.zip › antibiotics-2495080-supplementary.pdf]

Supplementary File S1: Antibiotic resistant trends of *A. baumannii.* AMI: amikacin, AMP: ampicillin, ATM: aztreonam, CAZ: ceftazidime, CIP: ciprofloxacin, COL: colistin, COT: co-trimoxazole (trimethoprim-sulfamethoxazole), CTX: cefotaxime, CXM: cefuroxime, ETP: ertapenem, FEP: cefepime, GEN: gentamycin, IMI: imipenem, MEM: meropenem, SAM: ampicillin-sulbactam, SPZ: sulperazone (cefoperazone-sulbactam), TET: tetracycline, TOB: tobramycin, TZP: tazocin (piperacillin-tazobactam).

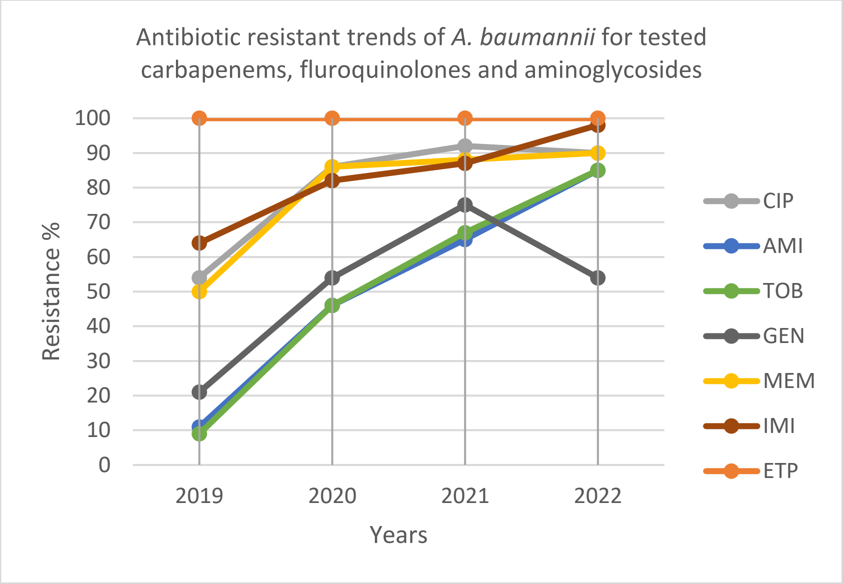

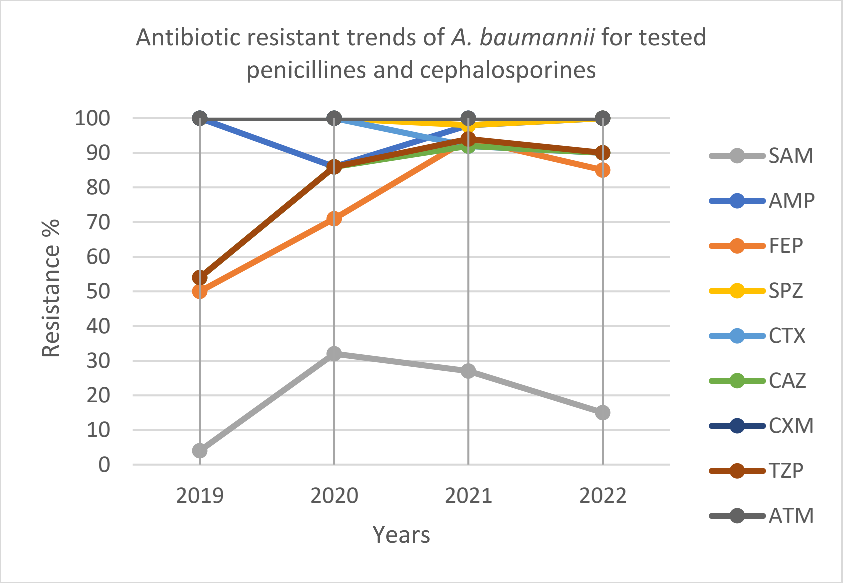

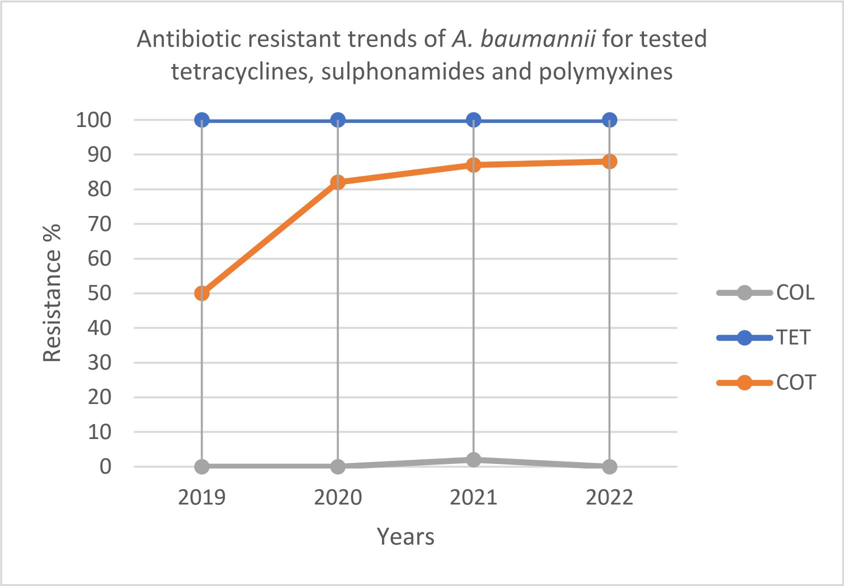
Antibiotic resistant trends of *A. baumannii* for tested tetracyclines, sulphonamides and polymyxines
Resistance %
100
90
80
70
60
50
40
30
20
10
0
2019
2020
2021
2022
Years
COL
TET
COT
